# Insights into Ultrasound Features and Risk Stratification Systems in Pediatric Patients with Thyroid Nodules

**DOI:** 10.3390/jimaging10080189

**Published:** 2024-08-05

**Authors:** Carla Gambale, José Vicente Rocha, Alessandro Prete, Elisa Minaldi, Rossella Elisei, Antonio Matrone

**Affiliations:** 1 Unit of Endocrinology, Department of Clinical and Experimental Medicine, Pisa University Hospital, 56124 Pisa, Italy; carla.gambale@phd.unipi.it (C.G.); alessandro.prete@phd.unipi.it (A.P.); elisa.minaldi@phd.unipi.it (E.M.); rossella.elisei@unipi.it (R.E.); 2 Serviço de Endocrinologia, Diabetes e Metabolismo, Unidade Local de Saúde Santa Maria, 1649-028 Lisboa, Portugal; j.rocha1@campus.ul.pt

**Keywords:** thyroid nodules, neck ultrasound, malignancy, risk stratification systems, pediatric age, ultrasound features, thyroid carcinoma, management

## Abstract

Thyroid nodules in pediatric patients are less common than in adults but show a higher malignancy rate. Accordingly, the management of thyroid nodules in pediatric patients is more complex the younger the patient is, needing careful evaluation by physicians. In adult patients, specific ultrasound (US) features have been associated with an increased risk of malignancy (ROM) in thyroid nodules. Moreover, several US risk stratification systems (RSSs) combining the US features of the nodule were built to define the ROM. RSSs are developed for the adult population and their use has not been fully validated in pediatric patients. This study aimed to evaluate the available data about US features of thyroid nodules in pediatric patients and to provide a summary of the evidence regarding the performance of RSS in predicting malignancy. Moreover, insights into the management of thyroid nodules in pediatric patients will be provided.

## 1. Introduction

Thyroid nodules in pediatric patients are less common than in adults. However, either in pediatrics or in adults, the prevalence of thyroid nodules varies according to the method of detection, being 0.5% and 2–6% with palpation and 2% and 19–35% with ultrasound (US) in pediatrics and adults, respectively [1,2]. The introduction into clinical practice of neck US drastically increased the discovery of thyroid nodules in the general population, particularly if compared with palpation [3,4]. However, although most of the thyroid nodules occasionally discovered by neck US are not clinically relevant (small dimension, not suspicious for malignancy), an increase in the diagnosis of differentiated thyroid cancer (DTC) was highlighted [5]. Of note, particularly in pediatric patients, several non-thyroid conditions, including abscesses, lymphatic or vascular malformations, thyroglossal duct cysts, ectopic thymus, and tumors, can resemble thyroid nodules, making necessary the use of neck US [4]. The question of whether a potential link between a higher incidence of thyroid nodules in children and some clinical risk factors exists and remains controversial. Regarding risk factors, recently, a higher risk of having thyroid nodules in pediatric patients with a positive family history of thyroid nodules has been reported [6]. The same authors found that the incidence of thyroid nodules in pediatric patients with chronic lymphocytic thyroiditis is lower (4.8%) than those previously reported by an Italian multicenter study (31.5%) [7]. Despite these controversies about the incidence of thyroid nodules in childhood, the available data show that the malignancy rate of thyroid nodules is higher in pediatrics (22–26%) than in adults (5–10%) [8]. For this reason, a careful evaluation is needed in pediatric patients with thyroid nodules to assess the risk of malignancy, aiming to discriminate benign from malignant nodules. Additionally, other issues that need further clarification pertain to planning effective clinical management strategies aimed at minimizing both the rate of missed malignancies and the number of unnecessary fine needle aspiration (FNA) procedures. Thyroid US is nowadays the best and most used tool to evaluate the position, dimension, and features of thyroid nodules, even in pediatric patients. In adult patients, specific US features have been associated with an increased risk of malignancy in thyroid nodules, such as irregular margins, marked hypoechogenicity, microcalcifications, and a “taller than wide” shape [9]. Therefore, several US risk stratification systems (RSSs) have been built by scientific societies to help clinicians define the US risk of malignancy (ROM) of the thyroid nodules [10,11,12,13,14]. The RSSs aim to reduce variability and the improve inter-observer reproducibility of the description of ultrasound features and define the risk of malignancy of thyroid nodules. Furthermore, they facilitate communication between operators aiming to standardize clinical management and providing indications for performing FNA based on scores and dimensions [15]. The most used RSSs implemented in clinical practice are designed by the European Thyroid Association (European Thyroid Imaging and Reporting Data System (EU-TIRADS)) [10], American Thyroid Association (2015 ATA) [11], American College of Radiology (ACR-TIRADS) [13], American Association of Clinical Endocrinologists/American College of Endocrinology/Associazione Medici Endocrinologi (AACE/ACE/AME) [12], and Korean Society of Thyroid Radiology (K-TIRADS) [16]. RSSs can be divided into those considering the combination of US features of the nodule (“pattern-based”) and those assigning points to each ultrasound feature building a score (“point-based”) to define the ROM. Although the “point-based” systems apparently show the highest performance compared to “pattern-based”, currently no system has consistently shown superiority over the observer reproducibility of the scores, which remains comparable [17]. It is worth noting that these RSSs are designed for identifying papillary thyroid carcinoma (PTC), mainly in its classic or aggressive (i.e., tall cell, columnar cell, hobnail, etc.) variants (CV-PTC, AV-PTC), while their performance is less accurate both in medullary thyroid carcinoma (MTC) [18] and in the follicular variant of papillary thyroid carcinoma (FV-PTC) and follicular thyroid carcinoma (FTC) [19,20]. Moreover, RSSs were developed having the adult population as a reference and their use has not been fully validated in pediatric patients.

The present paper aims to assess the performance of the US features of thyroid nodules and RSSs in predicting malignancy in pediatric patients by reviewing the available literature. 

## 2. Ultrasound Features 

The accuracy of thyroid US in distinguishing between benign and malignant thyroid nodules is based either on specific ultrasound features or their combination. Several papers have evaluated the ROM of the US features of thyroid nodules in pediatric patients.

### 2.1. Nodule Dimension

The data about the association between nodule dimension and the ROM are quite well defined. Several papers [21,22,23] support the presence of a direct correlation between large nodule dimension and malignancy. Richman et al. [24], in a series of 404 nodules in 314 patients younger than 19 years, found that about one-third (33.8%) of 77 malignant nodules had dimensions≥ 30 mm compared to 21.4% of benign nodules (*p* = 0.04). In this study, in which the definition of benign or malignant was based on histology results after surgery or cytology results after FNA, most of the cases were PTC, with a higher rate of sclerosing diffuse variant, but also with some cases of FTC and MTC. Koltin et al. [22] in a smaller cohort of 27 pediatric patients, with a mean age of 13.1 years, demonstrated that a larger nodule dimension (>35 mm) was strongly associated with malignancy (*p* = 0.007). In this paper, all malignant cases (19/27—70.4%) were DTC (17 PTC and 2 FTC), while eight (29.6%) nodules were benign. The strength of this paper was that all thyroid nodules were surgically treated, so this certainly makes the definition of benign and malignant more accurate. Fornwalt et al. [23], analyzing 112 thyroid nodules in pediatric patients (mean age 14.3 years), defined the same cut-off of nodule dimension (>35 mm) as more frequently associated with malignancy. Gupta et al. [25] reported that in 125 pediatric patients (≤18 years) with a total of 136 nodules, the median dimension (29.5 mm) was higher in malignant nodules, all defined by cytology, than in benign ones (22.5 mm) (*p* = 0.004). Also, Cimbek et al. [21] found that in pediatric patients with a younger age compared to the previously described studies (mean 10.6 years), benign and malignant nodules differed in dimension: 125 benign nodules had a mean dimension of 6.5 mm compared to 6 malignant nodules with a mean dimension of 12.9 mm (*p* = 0.002). Moreover, 60% of malignant nodules had dimensions ≥ 10 mm compared to 15.5% of benign ones. However, in this study, the number of malignant nodules was very low (6/131 nodules—4.6%), and only a minority of the benign nodules (21/125—16.8%) were defined based on cytology and/or histology, while the remaining (104/125—83.2%) were considered benign only according to US results and clinical observation. Gannon et al. [26] analyzed 236 nodules in patients with ages **≤** 18 and found that nodule dimensions between 1 and 4 cm showed the highest probability of benignity (62%) compared with those <1 cm (32%) and >4 cm (5.7%). 

Conversely, some other studies failed to identify a clear association between a larger nodule dimension and malignancy. Canfarotta et al. [27] analyzed 46 patients (mean age 14.8 years) and found no differences in dimension when comparing 36 benign and 10 malignant nodules divided according to three categories of dimension (2–2.9, 3–3.9, and > 4 cm). This paper used the McGill score of [28] in the thyroid nodules of pediatric patients. However, since this score can be applied only for nodules measuring at least 2 cm, this could influence the comparison with other studies. Despite the low number of cases, this study demonstrated that a comprehensive clinical, radiologic, and pathologic scoring system may help in assessing the ROM in pediatric thyroid nodules.

### 2.2. Echogenicity

The sensitivity and specificity of echogenicity in predicting malignancy in thyroid nodules in pediatric patients, particularly hypoechogenicity, varies in the different papers. Hypoechogenicity showed low sensitivity (63%) and specificity (50.2%) in identifying malignancy in the study of Richman et al. [24]. Gannon et al. reported that having non-isoechoic thyroid nodules was associated with a sensitivity of 83.5% and a specificity of 52.6% for having thyroid carcinoma in pediatric patients [26]. Also, Goldfarb et al. [29] reported that echogenicity alone was not able to predict malignancy. Indeed, when analyzing the US features of thyroid nodules in 50 patients (mean age 17.4 years), the rate of hypoechogenicity, although more frequent in malignant nodules, was not different between malignant and benign nodules (51.9 vs. 36.4%—*p* = 0.335). Also, Buryk et al. [30], in 89 pediatric patients (median age 15.1.years), reported a high sensitivity (88%) and negative predictive value (85%) but, unfortunately, a low specificity (46%) and positive predictive value (50%) for nodule hypoechogenicity in predicting malignancy. 

### 2.3. Irregular Margins

The presence of irregular margins in predicting malignancy in thyroid nodules in pediatrics showed variable sensitivity (51.9–69.6%) [26,31] and specificity (86.4–94.1%) [24,31] according to the study considered, although the majority of studies agree in defining this US feature highly predictive of malignancy [22,32]. However, different from the majority of the papers, the study of Fornwalt et al. [23] did not find any association between irregular margins and malignancy. 

### 2.4. Shape

Interestingly, the nodular shape taller-than-wide showed a low sensitivity (21.2–26.4%) [24,26] but high specificity (89.7–92.3%) [24,26] in predicting malignancy in pediatric nodules. Yu et al. [33], in a series of 173 thyroid nodules in 152 pediatric patients (median age 16 years) that was recently published, reported an overall incidence of a taller-than-wide shape of 15.61%. However, this rate was significantly different between 69 benign and 104 malignant nodules (4.35 vs. 23.08%—*p* <0.01), mostly PTC. In this paper, again, the definition of malignancy was not uniform being based on FNA (19.65%) or histology after surgery (80.35%). Moreover, also these authors observed that the taller-than-wide shape had a high specificity (95.65%) but low sensitivity (23.08%) in predicting malignancy in pediatric patients. Polat et al. [34] confirmed the different incidence of the taller-than-wide shape between 100 benign and 5 malignant nodules (6% vs. 60%, respectively) in patients with a mean age of 11.4 years. Nevertheless, the small number of malignant nodules in this paper could explain the higher rate of the taller-than-wide shape compared with those reported by others. 

### 2.5. Calcifications

The current papers in the literature are quite in agreement about the role of calcifications, mainly microcalcifications, in predicting the malignancy of thyroid nodules in pediatric patients [21,22,23,24]. Particularly, Creo et al. [32] analyzing 112 pediatric patients (mean age 15.5 years) with a total of 145 thyroid nodules found that having microcalcifications showed a greater potency of association with malignancy. However, in a previous paper by Janus et al. [35], who analyzed US images of 18 patients with PTC and ages varying from 7 to 20 years, the findings in the US were heterogeneous. Indeed, the authors observed PTC without microcalcifications in 61% of the cases, supporting the evidence that the lack of microcalcifications did not exclude malignancy. A potential explanation for this difference is the difficulty in correctly describing the type of calcifications in peculiar settings in clinical practice, such as chronic lymphocytic thyroiditis that ranged from 6 to 64.5% [18,19,36], according to the different histology of the nodule. Indeed, by following the AACE/ACE-AME guidelines [12], the microcalcifications are tiny hyperechoic punctuate spots <1 mm, without posterior shadow, suggestive of malignancy and different from the more frequent and innocuous “comet tail” sign characterized by bright hyperechoic spots that are often associated with non-malignant nodules. Of note, some authors reported anecdotal cases in which also the presence of diffuse microcalcifications in the thyroid gland, even without evidence of nodules, can be considered a potential sign of malignancy [37,38]. 

### 2.6. Blood Flow at Color Doppler

Although blood flow at color Doppler is not a specific US feature associated with malignancy in adult patients, several papers have reported that an increased blood flow at color Doppler in pediatric patients is observed in malignant nodules. Buryk et al. [30] reported that increased blood flow at color Doppler showed a high sensitivity (80%) and negative predictive value (89%) but a low specificity (20%) and positive predictive value (15%) in predicting malignancy. Creo et al. [32] showed that increased blood flow at color Doppler positively correlated with malignancy. This evidence was indirectly confirmed by Gannon et al. [26], who showed that the absence of blood flow at color Doppler was strongly associated with benignity.

In Table 1, we report the sensitivity and specificity of the ultrasound features associated with thyroid nodule malignancy in pediatric patients. 

### 2.7. Suspicious Lymph Node Metastases

Regardless of thyroid nodule features, the presence of suspicious lymph node metastases of the central and/or latero-cervical compartment, detected at the neck US, increases the risk of malignancy [39]. US features of suspicious lymph node metastases are hypoechogenicity/cystic appearance, microcalcifications, peripheral vascularity, and round shape [2]. Therefore, in these cases, FNAC should be performed both in thyroid nodules and lymph nodes (with the evaluation of thyroglobulin on washing fluid) [15].

## 3. Risk Stratification Systems (RSSs)

RSSs, which combine the US features of thyroid nodules in predicting malignancy, are widely used in the adult population. However, several papers have also analyzed the diagnostic performance of different RSSs in pediatrics. 

### 3.1. 2015 ATA

Creo et al. [32] analyzed the performance of 2015 ATA in detecting malignancy in 112 pediatric patients with 145 thyroid nodules. Most of the nodules were classified in the high (56%) and intermediate (19%) suspicion categories, respectively. Of the 38 malignant nodules, 46% were classified in the high and 32% in the intermediate suspicion category. The authors found a high sensitivity (91%) but low specificity (54%) in predicting malignancy. Kim et al. [40] performed a meta-analysis including eight papers for a total of 1036 thyroid nodules in pediatric patients (mean/median age ranged from 11 to 16 years in the different studies): the pooled ROM for thyroid nodules classified in 2015 ATA for high (55.4%) and intermediate suspicion (34.2%) was comparable with adults. Conversely, the pooled ROM for the categories at low risk was higher in pediatric patients compared with adults. Similar findings are reported in a meta-analysis performed by Xing et al. [41] that included 19 papers evaluating the diagnostic performance of four RSSs (2015 ATA, ACR-TIRADS, EU-TIRADS, and K-TIRADS) in pediatric patients. The paper included 1927 patients with an age range from 0.9 to 22 years and 2263 nodules. Regarding the 2015 ATA, the pooled sensitivity and specificity in predicting malignancy were 84% for high suspicion and 55% for intermediate suspicion category. Martinez-Rios et al. [42] evaluated the performance of 2015 ATA and ACR-TIRADS in pediatric patients (mean age 13.6 years) with a total of 123 nodules (>1 cm). The benignity (*n* = 71) or malignancy (*n* = 52) was defined either with histology and/or cytology or with clinical and sonographic stability during a minimum 2-year follow-up without histologic or cytologic data. According to the 2015 ATA, the 52 malignant nodules were classified as high US suspicion in 69.2%, intermediate suspicion in 11.5%, and low/very low suspicion/benign in the remaining 13.4% of the cases. The rate of malignancy, mostly defined at histology a/o cytology, was 74% for the high suspicion category, decreasing to 43% in intermediate, 14% in low, 10% in very low, and 0% in benign. Lim-Dunham et al. [43] showed the highest malignancy rate in pediatric thyroid nodules classified in the 2015 ATA high suspicion category. Of note, none of the 12 thyroid cancers were classified into the low/intermediate suspicion category but a significant number (42.9%) of benign nodules were incorrectly classified as high suspicion. In a very recent paper, Yu et al. [33] assessed the performance of three RSSs (ACR-TIRADS, Chinese TIRADS, and 2015 ATA) in identifying malignancy and suggesting FNA indication in 173 thyroid nodules of patients ≤ 18 years. When evaluating the performance of 2015 ATA in identifying malignancy, sensitivity was 70.1%, specificity was 43.28%, and 2015 ATA showed the highest missed malignancy rate (50%) and unnecessary FNA rate (35.85%) compared with the other RSSs considered.

### 3.2. ACR-TIRADS

In a recent paper, Hess et al. [44] evaluated the performance of ACR-TIRADS in identifying malignancy in 208 thyroid nodules of 142 patients (range 3.1–18.8 years). When considering as a reference only the 74 patients receiving surgery, then with available histology, the ROM was 0% for TR1, 20% for TR2, 41.2% for TR3, 37.5% for TR4, and 72.2% for TR5. Moreover, the authors pooled their data with other already published pediatric cohorts from other institutions and analyzed the performance of ACR-TIRADS for a total of 1458 nodules. The ROM was 2.2% for TR1, 9.3% for TR2, 16.6% for TR3, 27% for TR4, and 76.5% for TR5. These results highlighted that ACR-TIRADS categories showed a higher ROM for the pediatric population compared with adults. However, most of the studies included in this analysis did not use a single method for defining malignancy but a combination of methods including, in addition to cytological and/or histological results, non-standardized clinical and ultrasound criteria such as the dimensional stability of the nodules over time. 

Different results were highlighted in the paper by Martinez-Rios et al. [42] in which malignant nodules were classified as TR4 in most of the cases (88.5%) and TR5 in only 11.5% of cases.

The meta-analysis performed by Kim et al. [40] also assessed ACR-TIRADS. The pooled ROM for the two higher suspicion categories (TR5—59.3% and TR 4—20.7%) was like adults. Conversely, the pooled ROM for the categories at low suspicion of malignancy was higher in pediatric patients compared with adults. Xing et al. [41] confirmed the good performance of ACR-TIRADS: the pooled sensitivity and specificity in predicting malignancy were 84% for TR4 and 61% for TR 5.

In the series of Richman et al. [45], 77 out of 404 nodules (19.1%) were malignant and the rate of malignancy in the nodules classified as ACR-TIRADS 5 was 74.2%, similar to that reported in adults. In another paper [46], the same authors analyzed 87 nodules with indeterminate cytology in 78 patients aged less than 19 years. They found that the TR5 category, according to ACR-TIRADS, was associated with a higher rate of malignancy compared to the other categories (80% vs. 42%, *p* = 0.002). 

In the paper by Yu et al. [33], ACR-TIRADS showed a sensitivity of 71.15% and a specificity of 73.91% in the ability to identify malignancy. The results of the missed malignancy rate (37.04%) and unnecessary FNA rate (19.57%) were better than the 2015 ATA.

The risk of malignancy in pediatric thyroid nodules seems to also remain non-negligible in the not suspicious/benign category, which is different from adults. Indeed, Scappaticcio et al. [47], although including a small number of patients (n = 36) and nodules (n = 41), found a higher risk of malignancy in the highest suspicion category of four RSSs (2015 ATA high suspicion, ACR-TIRADS TR5, EU-TIRADS 5, and K-TIRADS 5). Although the ROM in the categories at high suspicion was similar to those detected in the adult population, it was higher than in adults in the low suspicion and not suspicious/benign categories. Regarding ACR-TIRADS, the authors reported an ROM of 30% for TR3 and 20% for TR2, clearly higher than those reported for the same categories in the adults (5% for TR3 and < 2% for TR2).

### 3.3. EU-TIRADS

In their meta-analysis, Xing et al. [41] demonstrated that the EU-TIRADS 4 and 5 categories showed a pooled sensitivity of 78% and specificity of 48% in identifying malignancy, apparently having lower performance than the higher suspicion categories of ACR-TIRADS and 2015 ATA. Similarly, Tuli et al. [48], analyzing 200 nodules of pediatric patients younger than 18 years, found a lower diagnostic performance for EU-TIRADS in detecting malignancy compared to ACR-TIRADS. The rate of malignancy in the entire cohort, defined according to cytology, was 13%. The ROM, particularly between the highest suspicion categories EU-TIRADS 5 and ACR-TIRADS 5 (23.8% and 53.8%), was very different. Of note, in this series of pediatric thyroid nodules, the ROM in the high suspicion category was lower for EU-TIRADS (EU-TIRADS 5—23.8 vs. 26–87%) and higher for ACR-TIRADS (TR5—53.8% vs. >20%) if compared with the same category in the same RSS in the adult population. Scappaticcio et al. [47] reported a higher ROM in the not suspicious/benign category compared with those reported for the same categories in the adults also for EU-TIRADS. Indeed, the authors found an ROM of 27.3% for EU-TIRADS 3 and 12.5% for EU-TIRADS 2, compared to 2–4% for EU-TIRADS 3 and 0% for EU-TIRADS 2 in adults. 

### 3.4. K-TIRADS

The performance of K-TIRADS 4–5 in identifying malignancy was quite low (pooled sensitivity 64%, pooled specificity 84%) compared to other RSSs in the paper by Xing et al. [41]. However, this result could be influenced by the small number of studies analyzing K-TIRADS performance (n = 3).

Regarding K-TIRADS, which was built in 2016, this RSS showed higher sensitivity but lower specificity, with a higher number of potential unnecessary biopsies compared with other RSSs [49]. For this reason, an improved version of K-TIRADS was published in 2021, mainly characterized by an increase in the nodule biopsy cut-off dimension [14], with improved specificity and a lower unnecessary biopsy rate compared with the 2016 version. By using 2021 K-TIRADS, Kim et al. [50] demonstrated the higher diagnostic efficacy of the 2021 version compared to the 2016 version in pediatric patients. This improvement was particularly evident when a biopsy cut-off of 0.5 cm for K-TIRADS 5 and 1.0–1.5 cm for K-TIRADS 4 was used. 

The ROM in pediatrics was higher than adults in the categories at low suspicion of malignancy (K-TIRADS 3—27.3 vs. 3–15%) and not suspicious/benign (K-TIRADS 2—12.5 vs. 1–3%) in the paper by Scappaticcio et al. [47]. 

The ROM, according to the most used RSSs in clinical practice (i.e., 2015 ATA, ACR-TIRADS, and EU-TIRADS) for the different categories of suspicion, in pediatrics and adults, is reported in Table 2.

## 4. Discussion and Suggested Management

The management of thyroid nodules in pediatric patients is a challenge for physicians. This is mainly due to the complexity of the patients treated, particularly if they are younger than 10–12 years old. In this review, we focused our attention on the role of thyroid US in predicting malignancy, particularly papillary thyroid carcinomas, in pediatric thyroid nodules. It is worth noting that most of the reported studies did not have histology as the final reference, but also not cytology and, in several cases, particularly for the definition of benign nodules, only US features and lack of an increasing dimension over time have been considered. This makes it very difficult to estimate a real prevalence of benignity or malignancy in pediatric thyroid nodules according to the available literature.

**Figure 1:**
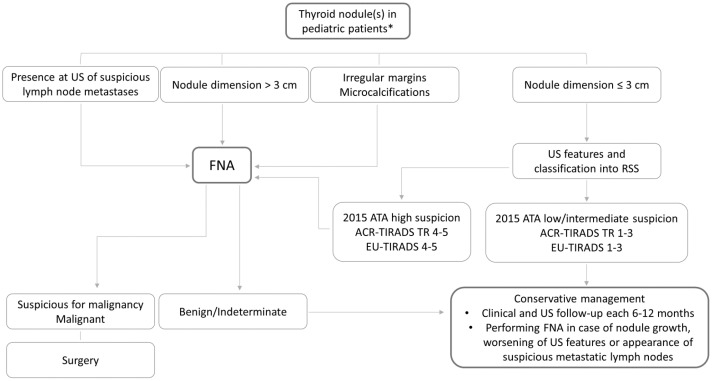
Suggested algorithm of thyroid nodule management in pediatric patients. * without pathologic calcitonin values and in the conditions of normal thyroid function.

Thyroid US is the diagnostic tool of choice in managing these patients; however, not all the evidence acquired in adults can be translated in pediatrics. To diagnose a malignant nodule, which is the final aim of the evaluation of a thyroid nodule, thyroid US is a key tool to suspect malignancy but also to avoid unnecessary FNA, which in pediatric patients could be difficult to perform, mainly because of the low collaboration of the young patient. Regardless of cases with detectable calcitonin, which should be evaluated in pediatric patients with thyroid nodules according to the suggestion of the guidelines [2], in the other cases the management should be guided by thyroid US. The available data show that in cases with large nodules (>30–35 mm), it is more frequent to detect malignancy [22,23,24]; therefore, FNA should be primarily reserved for these cases. Moreover, the presence of suspicious lymph node metastases, often present in PTC at the diagnosis in pediatrics, makes FNA mandatory. Beyond the large dimension, the other US features often associated with malignancy in thyroid nodules in adults, taken alone, do not have high sensitivity and specificity in identifying malignant nodules in pediatrics. Indeed, a taller-than-wide shape, which is considered a risk feature for the malignancy of thyroid nodules in several RSSs [10,11,12,13] in adults, has less significance in pediatrics. According to the data from the literature, greater attention should be given to the presence of irregular margins and calcifications [24,26,31,32]. These latter factors, microcalcifications should be carefully evaluated not only in thyroid nodules but also in the entire gland. Indeed, the sclerosing diffuse variant of PTC, which is more frequent in children than in adults, is usually associated with a diffuse spread of calcification throughout the gland, also without clearly detectable thyroid nodules. Also, the use of the main RSSs in identifying malignancy in pediatric patients has showed highly variable results. Indeed, the ROM is reliable and very high in the high suspicion US categories, while in the low and intermediate suspicion categories it can be higher than in adults [47]. This could be mainly related to the higher malignancy rate in the thyroid nodules of pediatric patients compared to that in adults (22–26%) [8]. However, despite this higher prevalence, it is worth noting that most of the cases are benign, particularly in the low intermediate suspicion US categories, and a conservative approach in these cases should be considered. The lack of prospective studies aiming to precisely define the US aspect of malignant thyroid nodules in pediatrics makes it difficult to draw definitive conclusions. According to the available data, we propose an algorithm for the management of newly discovered pediatric thyroid nodules, without pathologic calcitonin values and in conditions of normal thyroid function (Figure 1).

## 5. Conclusions

Thyroid nodules in children are rare but when present they generate great anxiety in parents and worry in the doctors who have to decide how to act. Thyroid US, which should necessarily include the evaluation of lymph nodes and particularly the latero-cervical compartment, plays the major role in deciding to perform FNA, which is the only diagnostic tool to identify the benign or malignant nature of the nodule. Some ultrasound features already identified as prognostic of malignancy in adults (i.e., greater dimensions, microcalcifications, irregular margins, hypoechogenicity, etc.), particularly if simultaneously detected, are also highly suggestive of malignancy in children and indicate the opportunity to perform FNA. It is worth noting that, by applying to children the same RSSs already used in adults, the risk of having cancer is like that of adults in the higher suspicion categories but higher in the lower suspicion categories. Despite this evidence, most of the thyroid nodules diagnosed in pediatric patients are benign; therefore, in those cases without clear evidence of worrisome US features, a careful but relaxed follow-up is advocated.

## Figures and Tables

**Table 1:** Sensitivity and specificity of the main US features of thyroid nodules in predicting malignancy in pediatric patients.

	Sensitivity	Specificity
Hypoechogenicity [20,21,23,27]	60.9–83.5%	20–52.6%
Irregular margins [21,23,27]	51.9–73.9%	45–94.1%
Taller-than-wide shape [21,23]	21.2–26.4%	89.7–92.3%
Microcalcifications [20,21,23,27]	58.4–89.8%	46–93.3%
Increased blood flow [20,23,27]	55.4–90.9%	25.9–53%

**Table 2:** ROM in pediatric compared to adult patients in the 3 main RSSs used in clinical practice.

		ROM (%)	
		Pediatrics	Adults
2015 ATA	Benign	0 [36,42,43]	<1
	Very low suspicion	0–10 [36,42,43]	<3
	Low suspicion	12.2–28.6 [36,42,43]	5–10
	Intermediate suspicion	12.5–43 [36,42,43]	10–20
ACR TIRADS	High suspicion	55.4–100 [36,42,43]	>70–90
	2	0–20 [35,36,40,41,43]	<2
	3	4.1–41.2 [35,36,40,41,43]	5
	4	12.5–37.5 [35,36,40,41,43]	5–20
	5	53.8–100 [35,36,40,41,43]	>20
EU-TIRADS	2	0–12.5 [40,43]	≅0
	3	8.3–27.3 [40,43]	2–4
	4	12.5–16.9 [40,43]	6–17
	5	23.8–100 [40,43]	26–87

## Data Availability

Not applicable.
